# *Pedicularis rostratospicata* subsp. *marsica* (*P.* Sect. *Rostratae*, Orobanchaceae), a New Subspecies from the Central Apennines (Italy)

**DOI:** 10.3390/plants12142614

**Published:** 2023-07-11

**Authors:** Fabio Conti, Christoph Oberprieler, Marco Dorfner, Erik Schabel, Fabrizio Bartolucci

**Affiliations:** 1 Floristic Research Center of the Apennine, University of Camerino—Gran Sasso Laga National Park, San Colombo, 67021 Barisciano, Italy; fabio.conti@unicam.it; 2 Evolutionary and Systematic Botany Group, Institute of Plant Biology, University of Regensburg, Universitätsstraße 31, D-93053 Regensburg, Germany; christoph.oberprieler@ur.de (C.O.); marco.dorfner@ur.de (M.D.); erik.schabel@stud.uni-regensburg.de (E.S.)

**Keywords:** Abruzzo, endemism, molecular phylogeny, National Park, nomenclature, taxonomy

## Abstract

The new subspecies *Pedicularis rostratospicata* subsp. *marsica* is here described based on morphological and molecular analyses. The new taxon is endemic to few localities of the Central Apennines within the Abruzzo, Lazio and Molise National Park (Central Italy). *Pedicularis rostratospicata* subsp. *marsica* can be distinguished from the other two currently accepted subspecies (subsp. *rostratospicata* and subsp. *helvetica*) by their taller stems, shorter petioles of basal and middle leaves, narrower blades of basal leaves, longer inflorescences with longer internodes and more flowers, and longer calyx lobes. Molecular analysis confirmed the autonomy of the new taxon. Furthermore, the conservation status assessment of the new subspecies according to IUCN categories and criteria is proposed and discussed, and an analytical key to the three subspecies of *P. rostratospicata* is presented.

## 1. Introduction

*Pedicularis* L. is the largest genus of Orobanchaceae Vent., with more than 600 species worldwide; it occurs mainly in arctic–alpine regions in the northern hemisphere [1,2]. There is no a recent worldwide revision of *Pedicularis*, or even a comprehensive phylogeny. In Italy, it is represented by 26 taxa (species and subspecies), and among these, only *P. elegans* Ten. is endemic to the Central and Southern Apennines [3,4,5].

*Pedicularis rostratospicata* Crantz belongs to a group of species that has usually been called *P.* sect. *Rostratae* Benth. (syn. *Rhyncholophae*), characterized by perennial herbs, with alternate floral and cauline leaves, and flowers with an upper lip that is more or less falcate, terminating in a distinct, usually long beak without teeth [6,7,8]. Currently, two subspecies are accepted: subsp. *rostratospicata* occurring mainly in the Central and Eastern Alps and in one locality of the Northern Apennines distributed in Germany, Helvetia, Austria, Slovenia and Italy [9] where it is recorded in Trentino-Alto Adige, Friuli-Venezia Giulia and Emilia-Romagna at Mt. Prado [4,10]; subsp. *helvetica* (Steininger) O.Schwarz occurring mainly in the Central and Western Alps distributed in France, Helvetia, Austria and Italy [9] where it is recorded in Valle d’Aosta, Piemonte, Lombardia, Trentino-Alto Adige and Liguria [4].

In Central Italy, *P. rostratospicata* is known only for few localities of the Abruzzo, Lazio and Molise National Park in Abruzzo administrative region [11,12]. According to Bruno and Bazzichelli [13], Bazzichelli and Furnari [14], Pignatti [15], and Conti and Bartolucci [16], this taxon deserves further study and actually does not fit among the known subspecies.

A morphological investigation and a molecular analysis have been carried out on living specimens and herbarium material coming from the Central Apennines (Italy) and the Alps, providing evidence of the differentiation between *P. rostratospicata* subsp. *rostratospicata*, *P. rostratospicata* subsp. *helvetica* and the Central Apennines populations. These results, and the disjunct and isolated geographical distribution of the populations occurring in the Central Apennines, allow us to refer them to a subspecies new to science, named *P. rostratospicata* subsp. *marsica*.

## 2. Materials and Methods

### 2.1. Plant Material

This study is based on field surveys, an extensive analysis of relevant literature, and an examination of herbarium specimens of *Pedicularis rostratospicata* kept in APP, BR, FI, GJO, H, JE, LJU, PESA, ROV, SPAL, WU, Z and ZT (acronyms follow Thiers [17]) and in the private herbaria of G. Pilsl (Herb. PILSL) and M. Bovio (Herb. Bovio). Morphological characteristics, recognized as taxonomically important in *Pedicularis* (see Table 1), were observed and measured under a Leica MZ16 stereoscopic microscope, using a digital calliper with 0.1 mm precision. Digital images of herbarium specimens from online databases were measured with IC MEASURE v.2.0.0.245 software. The distribution map of herbarium specimens examined was created using the free and open source QGIS ver. 3.30.0. software [18].

### 2.2. Morphometric Analyses

A total of 20 morphological characters were selected and scored for 15 dried individuals of *Pedicularis rostratospicata s.l.* (“MAR”) from three localities in the Central Apennines (Abruzzo: Mt. Marsicano, Mt. Petroso-Valle Cupella, and Serra delle Gravare), 19 individuals of *P. rostratospicata* subsp. *helvetica* (“HEL”) from several localities in the Western and Central Alps (Italy, Austria and Helvetia), and 21 individuals of *P. rostratospicata* subsp. *rostratospicata* (“ROS”) from the Eastern Alps (Italy, Austria and Slovenia), with a resulting dataset of 55 individuals × 20 variables. Of the morphological characters studied, 18 were quantitative and 2 were qualitative (Table 1). The quantitative and qualitative data were subjected to a principal coordinate analysis (PCoA) and cluster analysis (CA) using the average linkage method (UPGMA). The similarity matrix was calculated using the Gower coefficient, which is suitable for mixed data [19]. To test the current taxonomic hypothesis, we applied jackknifed linear discriminant analysis (LDA) with the individuals being a priori assigned to the three taxa based on morphological features. The multivariate analyses were carried out with the PAST v.4.11 software package [20].

Each quantitative continuous character was also subjected to univariate analysis (a Kruskal–Wallis test with Bonferroni corrections for multiple comparisons) with SPSS v.25 software [21]. Furthermore, the variability of the analyzed morphological characteristics was described using standard statistical parameters (mean, standard deviation, minimum, maximum, and 25th and 75th percentiles).

### 2.3. AFLPseq Fingerprinting

Genomic DNA for genetic fingerprinting was extracted in accordance with the CTAB DNA extraction protocol of Doyle and Dickson [22] and Doyle and Doyle [23]. The AFLPseq fingerprinting method was proposed by Dorfner et al. [24] and combines the genome complexity-reducing AFLP approach [25] with the next-generation sequencing (NGS) of resulting AFLP bands using the Nanopore sequencer MinION from Oxford Nanopore Technologies (Oxford, United Kingdom). It provides sequence and single-nucleotide polymorphism (SNP) information for hundreds of anonymous loci from across the whole genome and could be used for population genetic, phylogenetic, and species delimitation studies. It is suited for both well-preserved DNA from silica-gel dried leaf material and degraded DNA from herbarium specimens.

The present AFLPseq study comprised 11 *Pedicularis* accessions (Table 2), either recently collected, silica gel dried material (one accession of *P. rostratospicata* subsp. *helvetica* from Valle d’Aosta administrative region and three accessions of *P. rostratospicata* subsp. *rostratospicata* from Trentino-Alto Adige administrative region) or herbarium material housed in APP. The AFLPseq procedure followed the protocol given in Dorfner et al.’s work [24] with the following modifications. In the restriction ligation step, we used a double-digestion procedure with restriction enzymes MseI and EcoRI. After the ligation of MseI and EcoRI adapters (MseI adapter: 5′-GACGATGAGTCCTGAG-3′ + 5′-TACTCAGGACTCAT-3′; EcoRI adapter: 5′-CTCGTAGACTGCGTACC-3′ + 5′-AATTGGTACGCAGTCTAC-3′), we continued with the AFLP genome reduction protocol using primers with 1bp overhangs (MseI-C: 5′-GATGAGTCCTGAGTAAC-3′; EcoRI-A: 5′-GACTGCGTACCAATTCA) in the pre-selective amplification step and in the selective amplification step with additional 1bp (EcoRI 5′-GACTGCGTACCAATTCAA-3′) or 2bp-overhangs (MseI 5′-GATGAGTCCTGAGTAACTG-3′), respectively. The two primers used in the latter amplification step, however, were additionally tailored to include Nanopore barcode adapter sequences at the 5′ end of the primers (Mse_CTG_Nanopore_fw: 5′-TTTCTGTTGGTGCTGATATTGCGATGAGTCCTGAGTAACTG-3′; Eco_AA_Nanopore_rv: 5′-ACTTGCCTGTCGCTCTATCTTCGACTCCGTACCAATTCAA-3′), as suggested in the ‘Ligation sequencing amplicons—PCR barcoding (SQK-LSK109 with EXP-PBC001)’ protocol by Oxford Nanopore Technologies, substituting a subsequent ligation of the Nanopore barcode adapter with an additional barcoding PCR. To ensure specific binding with long and tailed primers, a two-step variation of the preselective PCR was conducted (94 °C for 2 min; followed by 30 cycles of 94 °C for 20 s and 72 °C for 2 min; and a final step at 72 °C for 2 min). To every 2 µL of a 1:10 diluted preselective PCR product, 5 µL of Taq DNA Polymerase Master Mix RED, 0.25 µL of each 10 µM tailed selective primer, and 2.5 µL of H_2_O were added. After the selective PCR, the length of the fragments ranged from 200 to 700 bp. All subsequent steps (Nanopore barcode PCR, sample multiplexing, size selection, and preparation of Nanopore sequencing library) followed those of Dorfner et al. [24]. The resulting library was sequenced with MinION using three Flongle flow cells. 

For the bioinformatic analysis of the Nanopore read data, we employed a simplified version of the SLANG pipeline [24], developed specifically for assembling and orthologizing loci from Nanopore reads while also facilitating variant calling. In our modified approach, we deviated from the original SLANG pipeline by clustering all reads of all OTUs (Operational Taxonomic Units) simultaneously using a single VSEARCH v.2.15.0 [26] clustering step. The clustering threshold was set at 0.85, indicating a high level of sequence similarity required for clustering, while also accounting for variation and Nanopore sequencing errors. This new approach eliminated the need for the within-sample clustering of individual OTUs, as well as among-sample clustering for locus orthologization, as all OTU reads were already orthologized via read clustering during the initial step of this modified approach.

For variant identification, we adopted the reference-based variant-calling approach of SLANG, albeit with the modification that allows all reads from an OTU to be mapped simultaneously to all consensus sequences, deviating from the original methodology, where only reads from the respective cluster were allowed to map to their respective reference sequence. This adjustment was implemented to minimize missing data and enhance data completeness. For mapping reads to a reference sequence, MINIMAP2 v.2.17 [27] was implemented. Subsequent variant extraction was employed in SLANG through BCFTOOLS [28]. Variants were considered valid if their frequency at the corresponding alignment position was equal to or greater than 30%, enabling the detection of heterozygosity in diploid individuals. Additionally, a minimum read depth of 5 for that position was required to ensure robust variant calling. Variants passing the filtering criteria were exported as a SNP alignment from the resulting VCF file through a custom script. Finally, we utilized the Python package PYCKMEANS (accessible at https://github.com/TankredO/pyckmeans; accessed on 6 June 2023) to compute Jukes–Cantor genetic distances and subsequently perform a principal coordinate analysis (PCoA) to visualize the relationships among the *Pedicularis* accessions.

## 3. Results and Discussion

### 3.1. Morphometric Analyses

**Figure 1:**
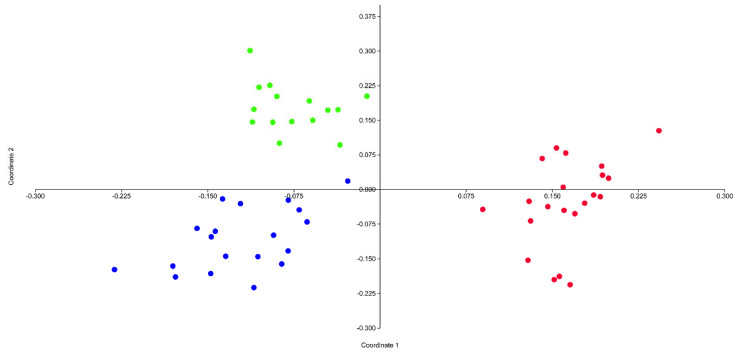
PCoA based on the 20 morphological characters measured in *Pedicularis rostratospicata* populations. Green dots represent individuals from the Central Apennines (MAR), blue dots represent individuals from the Central and Western Alps (HEL), and red dots represent individuals from the Eastern Alps (ROS).

**Figure 2:**
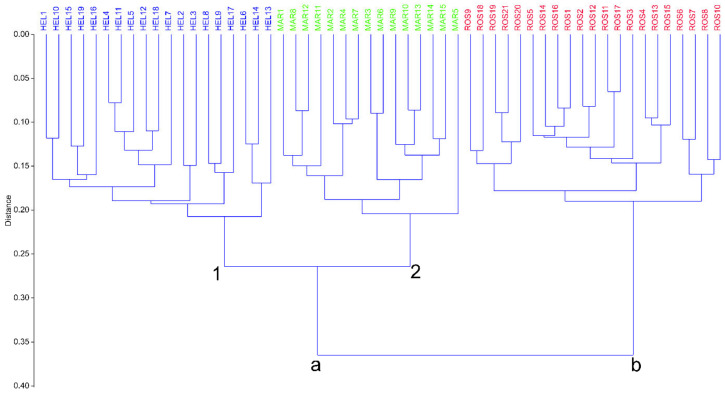
Hierarchical clustering of individuals of *Pedicularis rostratospicata* (ROS (Eastern Alps), HEL (Central and Western Alps), and MAR (Central Apennines)) using the Gower similarity index and the UPGMA cluster algorithm. The cophenetic correlation coefficient is 0.861.

**Figure 3:**
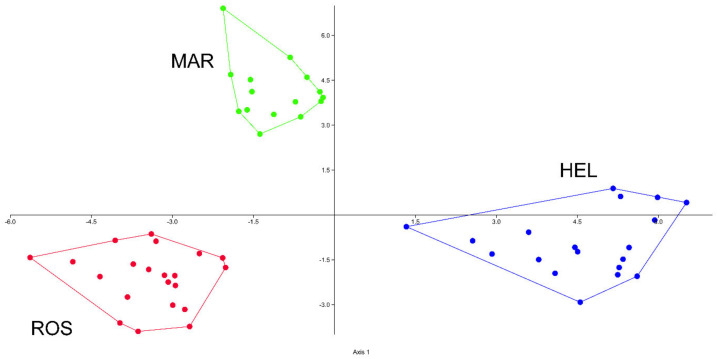
Linear discriminant analysis based on 17 quantitative continuous characters (ROS (Eastern Alps), HEL (Central and Western Alps), and MAR (Central Apennines)).

The first two axes of the PCoA explain 55% of the total variance. The scatterplot shows on the first two axes a clear separation of the three taxa (MAR, HEL, and ROS) involved in this study, and no overlapping areas among individuals were found (Figure 1). The UPGMA dendogram (Figure 2) shows two main clusters (“a” and “b”): cluster “a” composed of all the specimens of ROS, and cluster b composed of specimens of HEL and MAR. The latter cluster is further separated into two well-defined subclusters; “1” includes all the specimens referable to MAR and “2” includes all the specimens of HEL. Discriminant analysis resulted in 100% (jackknifed) correct classification of individuals a priori attributed to the three taxa (Figure 3). No overlap between individuals of MAR, ROS, and HEL was found, showing a clear distinction between these units.

**Figure 4:**
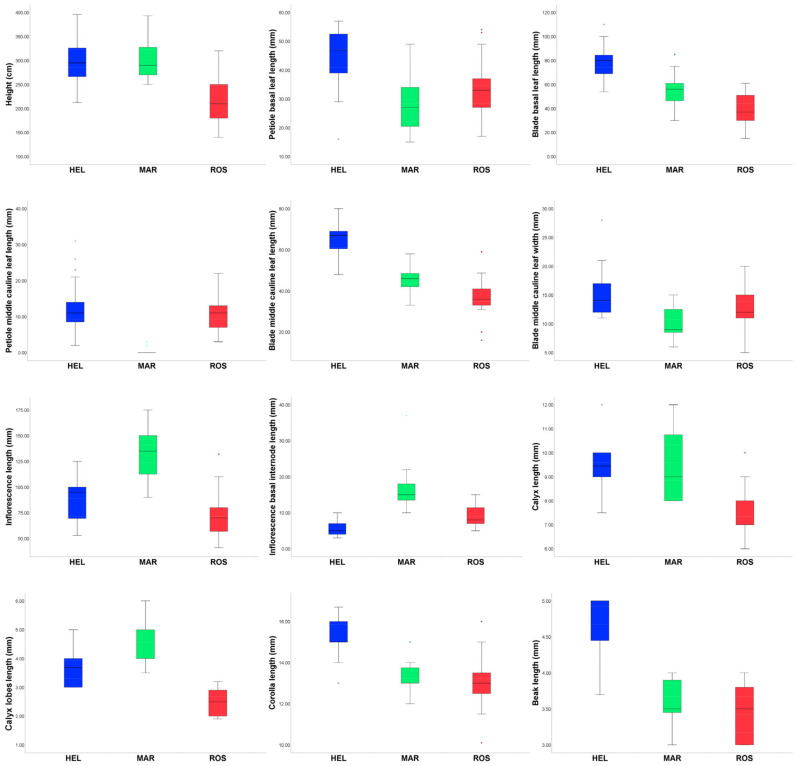
Boxplots showing those statistically significant morphological characters among the three taxa; ROS (Eastern Alps), HEL (Central and Western Alps), and MAR (Central Apennines). The outlined central box depicts middle 50% of data, extending from the 25th and 75th percentiles, and the horizontal bar is the median. Ends of vertical lines (or “whiskers”) indicate minimum and maximum data values, unless outliers are present, in which case whiskers extend to a maximum of 1.5 times the inter-quartile range. Circles indicate outliers.

The results of the univariate analysis of continuous characters are summarized in Table 3 and depicted in Figure 4. Based on the Kruskal–Wallis test, we conclude that at least one taxon is different in terms of the following characters (*p* < 0.01): H, PBLL, BBLL, PMLL, BMLL, BMLW, IL, IBIL, CL, CLL, COL, and NL. The states of three characters (H, CL, and SL) are significantly different between the ROS and the other two taxa (*p* < 0.01). The states of five characters (PBLL, BBLL, BMLL, CL, and NL) are significantly different between HEL and the other two taxa (*p* < 0.01). The states of three characters (PMLL, IL, and IBIL) are significantly different between MAR and the other two taxa (*p* < 0.01). In addition, the state of BMLW is significantly different between MAR and HEL.

Morphological analyses provide evidence that the Apennines populations of *P. rostratospicata* should be regarded as a new taxon, endemic to Central Italy. In addition, the study of the specimens collected on Mt. Prado (Emilia-Romagna administrative region) housed in FI and SPAL allowed us to review and refer to them as *P. rostratospicata* subsp. *helvetica*, as previously and correctly reported by Alessandrini et al. [29]. In subsequent papers [4,30], based on the indications from A. Alessandrini, however, it has been attributed by mistake to *P. rostratospicata* subsp. *rostratospicata*.

### 3.2. AFLPseq Fingerprinting

**Figure 5:**
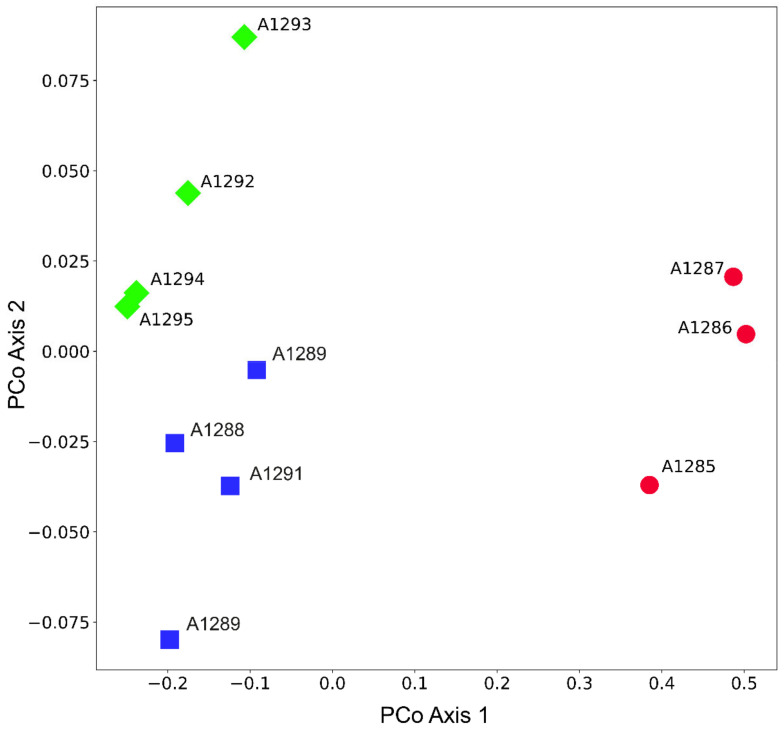
Ordination of a principal coordinates analysis (PCoA) of 11 OTUs of *Pedicularis rostratospicata* based on pairwise Jukes–Cantor distances calculated from AFLPseq fingerprint data (320 orthologous loci containing 3139 SNPs). While the first axis (PCo axis 1) explains 73.2% of the total variation, the second one accounts for 4.5%. Representatives of *P. rostratospicata* subsp. *rostratospicata* (ROS) are in red, those of subsp. *helvetica* (HEL) are in blue, and those of Apennines populations are in green (MAR).

In total, 194,212 reads and 68.66 Mbp were sequenced for the 11 *Pedicularis* accessions. After read preprocessing, 110,374 reads with lengths between 100 bp and 700 bp passed the Q5 quality filter. With the modified SLANG pipeline at a cluster threshold of 0.85, 320 orthologous loci were inferred, containing 3139 SNPs. After the calculation of Jukes–Cantor distances, the resulting PCoA plot was received (Figure 5), indicating a clear separation of *P. rostratospicata* subsp. *rostratospicata* (A1285–A1287) from other accessions through PCo axis 1 (explaining 73.2% of the total variation), while accessions from *P. rostratospicata* subsp. *helvetica* (A1288–A1291) and those from the Central Apennines (A1292–A1295) were separated on PCo axis 2 (explaining only 4.5% of the total variation). These results match the multivariate analysis of morphological characters in the study group (Figure 1 and Figure 2), where *P. rostratospicata* subsp. *rostratospicata* is well-separated from the other two taxa, while these show overlapping variation in some of the surveyed characters (Table 2, Figure 4).

Following Oberprieler [31] in his argumentation scheme on conceptualizing species rank decisions (the ‘Wettstein tesseract’), the present situation of genetic and morphological variation in the *P. rostratospicata* assembly may be best tackled in a taxonomic treatment either accepting three allopatric subspecies of a single species or dividing it into two species with *P. rostratospicata* subsp. *rostratospicata* on the one hand and subsp. *helvetica* plus the Central Apennines (Abruzzo) populations on the other. Despite the strong genetic and morphological separation of *P. rostratospicata* subsp. *rostratospicata* and subsp. *helvetica* seen in the present analyses, the lack of sympatry (mainly in the Eastern Alps for the former, Western and Central Alps for the latter) and the seemingly similar ecology of the two taxa (both being centered in the *Caricion ferrugineae* assembly showing the same substrate, precipitation and elevation niche and the same flowering time; [32]) argues for the subspecies rank for the two taxa as long as nothing is known about the strength of reproductive barriers between the two (code ‘1101’ in Figure 2 of Oberprieler 2023 [31]). On the other hand, the morphological distinctness (at least in multivariate analyses) of the allopatrically distributed *P. rostratospicata* subsp. *helvetica* and Central Apennines (Abruzzo) populations of the species, along with the only marginal differentiation in genetic respects, is also best captured taxonomically at the subspecific rank (code ‘0101’ or ‘0111’ in Figure 2 of Oberprieler 2023 [31]).

## 4. Taxonomic Treatment

**Figure 6:**
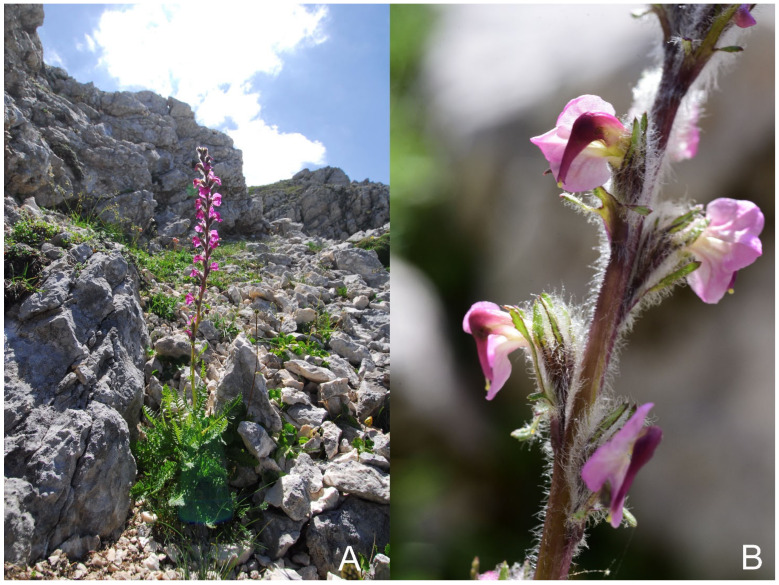
*Pedicularis rostratospicata* subsp. *marsica* F.Conti & Bartolucci (Italy, Abruzzo, Mt. Marsicano (**A**) and Valle Cupella (**B**), photos by F. Conti). (**A**) Habitat and flowering plant; (**B**) inflorescence and flowers.

***Pedicularis rostratospicata*** subsp. ***marsica*** F.Conti & Bartolucci, subsp. nov. (Figure 6).

**Figure 7:**
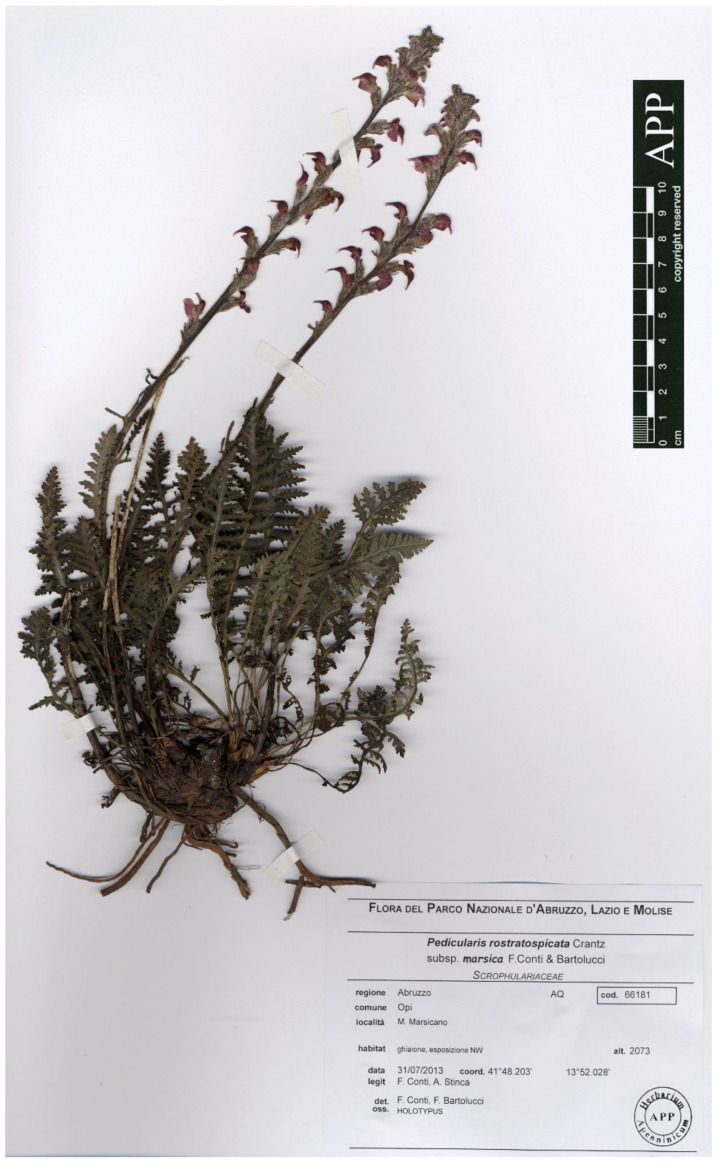
Holotype of *Pedicularis rostratospicata* subsp. *marsica* F.Conti & Bartolucci (APP No. 66181 and 66059, reproduced with permission from the Herbarium, Centro Ricerche Floristiche dell’Appennino, Italy).

Type: Italy. Abruzzo, M. Marsicano (Opi, L’Aquila), ghiaione, esposizione NW, 2073 m, 31 July 2013, *F. Conti, A. Stinca s.n.* (holotype APP No. 66181, Figure 7; isotypes APP No. 66176–66180).

**Figure 8:**
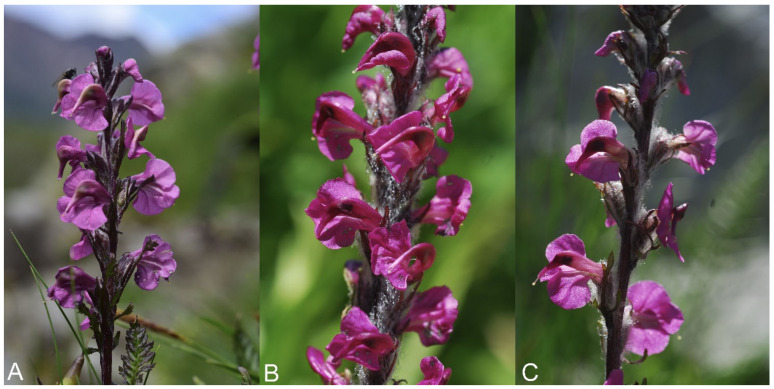
Comparison of *Pedicularis rostratospicata* subspecies: (**A**) *P. rostratospicata* subsp. *rostratospicata* from Val di Fleres locality (Italy, Trentino-Alto Adige, photo by F. Conti); (**B**) *P. rostratospicata* subsp. *helvetica* from Mt. Bianco locality (Italy, Valle d’Aosta, photo by F. Conti); (**C**) *P. rostratospicata* subsp. *marsica* from Mt. Petroso (Italy, Abruzzo, photo by F. Conti).

Diagnosis: *Pedicularis rostratospicata* subsp. *marsica* is distinguished from the other subspecies by its taller stems, shorter petioles in basal and middle leaves, narrower blades in basal leaves, longer inflorescences with longer internodes and more flowers, and longer calyx lobes (Figure 8).

Description: Perennial herbs, (25–)27–32.75(–39.3) cm tall, drying slightly black; stems ascending or erect, glabrescent in the basal part to shortly hairy in two rows to more densely arachnoid in the upper part. Leaves lanceolate, pinnatisect with pinnatifid segments. Basal leaves usually glabrous to more rarely sparsely hairy, petiole (15–)20.5–34(–49) mm long, blade (30–)46.5–61(–85) mm long × (10–)14–20(–25) mm wide. Cauline leaves alternate, the middle ones glabrous, with petiole 0–3 mm long, blade (33–)42–48.5(–58) mm long × (6–)8.5–12.5(–15) mm wide, the upper ones with short hairs along the midvein in the adaxial blade, with petiole 0–2 mm long, blade (9–)20–31(–41) mm long × (2–)3.5–6.5(–9) mm wide. Inflorescences racemose with short pedicels (90–)112.5–150(–175) mm long; internodes long, the basal ones (10–)13.5–18(–37) mm long, flowers (16–)20–25(–29). Bracts hairy with three linear lanceolate main lobes, (8–)14–20(–39) mm long. Calyx densely arachnoid tomentose, 8–10.75(–12) mm long; calyx lobes (3.5–)4–5(–6) mm long, in lower toothed flowers. Corolla rose, (12–)13–13.87(–15) mm long; beak purplish-red, (3–)3.45–3.9(–4) mm long; lower lip (11–)12–14(–16) mm wide, with three unequal lobes. Four filaments, glabrous, two longer at ca. 9 mm and two shorter at ca. 8 mm long, inserted at 2/3 of corolla tube. Capsule lanceolate-oblong, glabrous, 10–11 mm × 5–6 mm. Seeds ovoid, ca. 3.5 mm long.

Etimology: *Pedicularis rostratospicata* subsp. *marsica* is named after “Marsica”, a territory inhabited by the Marsi, an Italic people of ancient Italy.

Habitat: Ledges, screes in alpine belt from 1900 to 2100 m a.s.l., subject to long snow cover.

Phenology: Flowering from the end of June to beginning of August, fruiting from the end of July to August.

**Figure 9:**
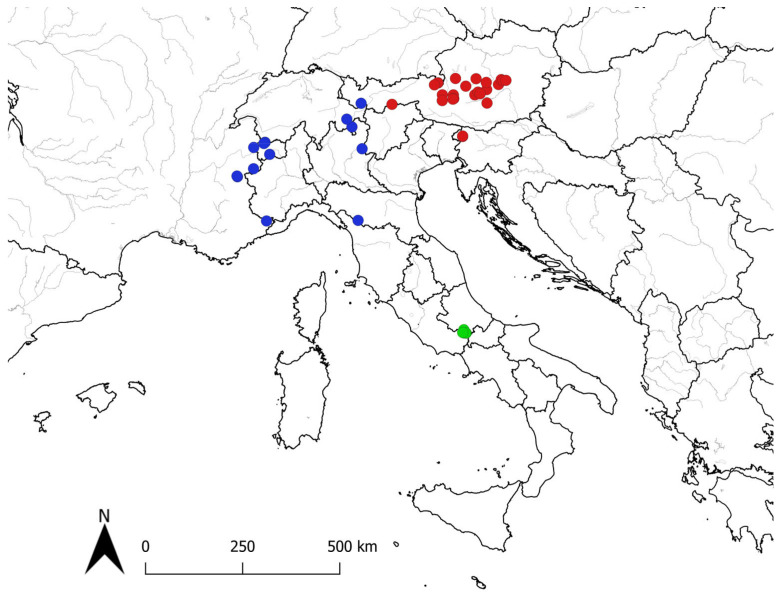
Distribution map of *Pedicularis rostratospicata* according to the herbarium materials studied: subsp. *rostratospicata* (red dots), subsp. *helvetica* (blue dots), and subsp. *marsica* (green dots).

Distribution: Endemic to the National Park of Abruzzo, Lazio and Molise (SAC IT7110205 “Parco Nazionale d’Abruzzo”) in Abruzzo administrative region distributed at Forca Resuni, Mt. Petroso, Valle Cupella, Serra delle Gravare, Mt. Marsicano [11,12,13,14,16,33]. The geographic distribution of *P. rostratospicata* based on the herbarium specimens examined is shown in Figure 9.

Conservation status: *Pedicularis rostratospicata* subsp. *marsica* is known to be in only one location (five subpopulations) within the NATURA 2000 network in SAC IT7110205 “Parco Nazionale d’Abruzzo”. The extent of occurrence (EOO) is 35,374 km^2^, and the area of occupancy (AOO) is 20 km^2^ (cell grid 2 × 2 km), as calculated with the GeoCAT (Geospatial Conservation Assessment Tool) software [34]. The main estimated threats include global warming, which favors the arrival of more thermophilous competitive species, and restricted range, which causes a greater risk of extinction in the event of disease or pest attacks. We have no data to evaluate whether or not the population is in decline and whether or not extreme fluctuations occur. According to IUCN criteria [35], the species is assessed to be near-threatened (NT).

### 4.1. List of the Specimens Examined

***Pedicularis rostratospicata*** Crantz subsp. ***rostratospicata***: **Austria**. Fleischrotes Läusekraut, Hochbwart bei Oberwölz, Urgebirge, 1900 m, 18 July 1929, *G. Genta s.n.* (GJO 0060417); Auf dem Kamme des Vordernberger Reichenstein in Obersteiermark, 26 July 1900, *K. Lorenz s.n.* (GJO No. 0060398); Schafhalssattel, 12 June 1925, *R. Wagner s.n.* (GJO 0060422); Eisenerzer-Alpen, am Eisenerzer Reichenstein., 21 June 1938, *K. Mecenovic s.n.* (GJO No. 0060408); Steiermark, Hochschwabgebiet, Edelsteig, auf Matten., alt. 1750 m-2277 m, 4 July 1950, *K. Mecenovic s.n.* (GJO 0060376); Obersteiermark: Hochmölbing, Vorgipfel-Anstieg, 21 July 1954, *K. Mecenovic s.n.* (GJO 0060379); Oberstmk.: Gesäuse-Alpen, Kalbling-Kar, 6 August 1954, *K. Mecenovic s.n.* (GJO No. 0060381); Niedere Tauern, Wölzer-Tauern, auf der Weißen Wand des Hohenwart bei Oberwölz (Ausgangapunkt: Schöttel-Jagdhütte!) auf kalk, 16 July 1960, *K. Mecenovic s.n.* (GJO No. 0060407); Steiermark: Neumarktersattel-Gebiet, am Furterteich bei Marianhof, Uferregion, Schilfzone, 18 June 1963, *K. Mecenovic s.n.* (GJO No. 0060396); Ostalpen, Nördl. Kalkalpen, Hochschwabgebiet, Schönleiten 1750 m, Rasen, 21 June 1957, *E. Habeler s.n.* (GJO 0060380); Niedere Tauern, Wölzer Tauern: Hang d Jauriskampel in Alpenmatten b. etwa 1950 m, 5 July 1976, *H. Melzer s.n.* (GJO 0060377); Steiermark; Wölzer Tauern, am Steilhang unter dem Kleinhansel bei Pusterwald in etwa 2100 m in Alpenmatten zahlreich, 12 July 1978, *H. Melzer s.n.* (GJO No. 0060378); Pongau, Radstädter Tauern, Kleinarltal, Aufstieg zum Tappenkar ca. 400 m vor dem See, 15 July 1979, *P. Pilsl 8745/4* (Herb. PILSL No. 1132); Fimbertal, in die Löcher, Almweiden, alt. 2100 m, 25 July 1980, H. Wittmann, *P. Pilsl s.n.* (Herb. PILSL No. 1829); Salzburg, Tennengau, Osterhorngruppe, Postalm-Gebiet, zwischen Braunedlkogel und Schmalzlager, Rinnbergsattel an der Landesgrenze zu Oberösterreich, gefestigter Dolomitschutt, 1540 msm, 47°37′55″ N 13°27′42″ E ± 0″, 21 June 2014, *P. Pilsl 8346/4* (Herb. PILSL No. 24004); Salzburg, Lungau, Radstädter Tauern, E vom Weißeck, E-Hänge des Kempen NE vom Mühlbachsee, kalkreiche alpine Rasen, 2160 msm, 47°09′53″ N 13°25′01″ E ± 2″, 18 July 2013, *P. Pilsl 8846/2* (Herb. PILSL No. 23825); Salzburg, Pinzgau, Steinernes Meer, E von Weißbach bei Lofer, Seehorn, Weg vom N-Grat Richtung Hochwies, alpine Rasen über Kalkgestein, 1930 msm, 47°31′15″ N 12°52′03″ E ± 3″, 27 July 2012, *P. Pilsl 8843/3* (Herb. PILSL No. 22865); Salzburg, Pinzgau, Steinernes Meer, E von Weißbach bei Lofer, Seehorn, Anstieg von der Kallbrunnalm oberhalb vom Seehornsee, alpine Rasen über Kalkgestein, 2000 msm, 47°31′01″ N 12°50′41″ E ± 5″, 27 July 2012, *P. Pilsl 8843/3* (Herb. PILSL No. 22834); Salzburg, Pinzgau, Rauriser Tal, NE von Rauris, Grubereck, E-Hang, von Felsbändern durchsetzte Rasen SE des Gipfels, 2110 msm, 47°14′42″ N 13°01′34″ E ± 2″, 13 July 2010, *P. Pilsl 8744/3* (Herb. PILSL No. 20772); Salzburg, Lungau, Murwinkel, Weißeck, Weg von der Sticklerhütte zur Riedingscharte, Almweiden im Bereich der Gräben N der Sticklerhütte, 2000 msm, 47°09′08″ N 13°23′16″ E ± 2″, 24 July 2009, *P. Pilsl 8846/1* (Herb. PILSL No. 19840); Salzburg, Pinzgau, Hohe Tauern, Raurisertal, Kalkbretterkopf, Grat Richtung Gaseiner Höhe (=S), 2300 msm, 47°06′50″ N 13°01′36″ E, 25 July 2008, *P. Pilsl 8844/3* (Herb. PILSL No. 18412); Salzburg, Pinzgau, Leoganger Steinberge, N Leogang, Weg von der Passauerhütte Richtung Hainfeldscharte N vom Mitterhorn, Kalkfelsrasen E der Hütte, 2000 msm, 11 June 2007, *P. Pilsl 8542/2* (Herb. PILSL No. 18144); Steiermark, Hochschwabgebiet, Mitteralm., 31 July 1955, *W. Maurer s.n.* (GJO No. 60423) [Steiermark, Hochschwabgruppe], [Bezirk Bruck-Mürzzuschlag, Gemeinde Thörl, Katastralgemeinde Fölz] Steiermark, Fölzalm, 1460 m [Quadrant 8457/1 (47°35′53,17″ N 15°11′22,19″ E ± 300 m] Kelchzipfel ganzrandig, behaart, 19 June 1966, *W. Maurer s.n.* (GJO No. 0085593); Steiermark, Dachsteingruppe, Bezirk Liezen, Gemeinde Aich, Katastralgemeinde Aich, am Stoderzinken, östlich etwas über der Steinerhütte, 1835 m, Quadrant 8458/2 (47°27′25,4″ N 13°49′02,7″ E ± 50 m, flachgründiger Rasen über Kalk, 3 August 2011, *K. Zernig 7590* (GJO No. 0073884); Niedere Tauern: Wölzer Tauern, Hohenwart, am steinigen Steilhang westlich des Fischsees längs einer Runse zahlreich bei etwa 1800 m Seehöhe, 13 August 1997, *H. Melzer s.n.* (GJO No. 0060421); Niedere Tauern: Wölzer Tauern, zwischen Plettentaler Joch und Sanderkogel im Rasen eines steilen Hanges gegen Hosten, 2000 m, Gneis mit etwas Marmor, 27 July 1995, *H. Melzer s.n.* (GJO No. 0060406); **Slovenia**. Julikske Alpe: pod Bogatinskim sealom. 1700 m, 17 July 1958, *T. Wraber 9478/1* (LJU No. 10040937); Julikske Alpe: In pratis inter pinos ubalpinas (*Pinus mugo*) planitiei altae Lepa Komna dictae. Solocalc. 1650 m, 5 July 1981, *T. Wraber s.n.* (H No. 1716561, LJU No. 111953); **Italy**. Trentino-Alto Adige: Val di Fleres, lungo il sentiero n. 8 per il Rifugio Calciati al Tribulaun (Colle Isarco), pendii rupestri, 2304 m, 14 July 2022, *F. Conti, F. Bartolucci s.n.* (APP Nos 66183–66188);

***Pedicularis rostratospicata*** subsp. ***helvetica*** (Steininger) O.Schwarz: **Helvetia**. La Baux, Grand St. Bernard, July 18 […], *J. Muret s.n.* (JE No. 00017632); Bernina, in […] Graubünden, August 1846, *Vulpius s.n.* (JE 00017631); Sommet de l’Albula, entre l’hospice et l’auberge, Grisons, end of July 1873, *Th. Brown s.n.* (H No. 1421035); Gr. St. Bernhard, 2300 m 18 July 1875, *J.F. Lerch s.n.* (JE No. 00017630); Gr. St. Bernhard, July 1890, *G. Müller s.n.* (ZT 00175344); **Austria**. Tirol, Fimbertal, Löcher, Almweiden, 2100 m, 25 July 1980, *H. Wittmann, P. Pilsl 9027/[…]* (Herb. PILSL No. 001829); **France**. Fontanalbe, Tende (A.-M.) Fr., sous-bois, 2100 m, 11 July 1986, *J. Bouharmont s.n.* (BR barcode BR00000361100414); Gazons humides du calcaire blanc et gypseux à Rivers sur le Mont Cenis (Savoie), June 1847, *A. Huguenin s.n.* (JE 00017629); Le Lautaret, prairie (2000–2300 m), 6 August 1910, *A. Faure s.n.* (H No. 1421066); Provence Alpes-Côte d’Azur, Cottische Alpen, Département Hautes Alpes, Arrondissement Briançon, Gemeinde Le Monêtier-les-Bains, rund 26 km NW Briançon, zwischen dem Col du Lautaret und dem Col du Galibier and der Passstraße im Vallon de Roche Noire unter dem Gipfel Pic-des-Trois-Evéchés, 2350 m (45°03′06.2″ N 6°23′19.5″ E ± 100 m, alpiner Rasen, 8 July 2014, *K. Zernig 9296* (GJO No. 0073091); **Italy**. Valle d’Aosta: Val Veny, pascoli sotto il Colle di Chavannes, 2170 m, 2 August 1982, *M. Bovio s.n.* (Herb. Bovio No. 173); Vallone del Gran San Bernardo, lungo la statale per il Colle del Gran S. Bernardo (Aosta), pascolo, 2275 m, 5 August 2021, *M. Bovio*, *M. Broglio*, *G. Ganz s.n.* (APP No. 66182); Val Veny (Courmayeur), 15 July 2014, *F. Conti, F. Bartolucci s.n*. (APP Nos 65463, 65465, 65466); Alpi Camoniche, pascoli nel versante S del M. Mattoni, tra Malga Bazenina e Passo Cadino, 2000–2100 msm, suolo calcareo, humus ± abbondante, 3 July 1986, *A. Brilli-Cattarini, L. Gubellini s.n.* (PESA); Alpe Pralognan-Alpe Ervillières 7 Km NE Cogne (AO), 2480 m, rasen, 2 July 1979, *R. Lais Wallisellen s.n.* (Z barcode Z-000206175); Emilia-Romagna: M. Prado, anticima nord, praterie, 1900–1950 m, 5 July 1981, *A. Alessandrini s.n.* (FI); Antic. di M. Prado (Ligonchio), vaccinieto, 1850 m, 16 June 2001, *G. Branchetti s.n.* (SPAL No. 1044);

***Pedicularis rostratospicata*** subsp. ***marsica*** F. Conti & Bartolucci: Italy. Abruzzo, M. Marsicano (Opi, L’Aquila), ghiaione, esposizione NW, 2073 m, 31 July 2013, *F. Conti, A. Stinca s.n.* (Holotype APP No. 66181, Isotypes APP Nos 66176–66180); Paratypes: Valle Cupella, Barrea, pendici del M. Petroso (Barrea), piccole zone di erosione, 1900–2000 m, 1 August 1997, *F. Conti s.n.* (APP No. 32730); M. Marsicano (Opi), 41°48.203′ N 13°52.028′ E, Ghiaione, Esp. NW, 2073 m, 30 July 2003, *F. Conti, D. Tinti, L. Eusepi s.n.* (APP No. 34905); tra M. Irto e M. S. Nicola, Serra delle Gravare (OPI), 6 August 2010, *F. Conti, G. Serafini s.n.* (APP No. 48238); Pietre Rosse (Villetta Barrea), 1750–1850 m, 21 July 2010, *F. Conti, G. Serafini s.n.* (APP No. 49268); M. Marsicano, presso la vetta, canalone subito sotto la cresta (Opi), 3 July 1987, *F. Conti s.n.* (APP No. 49706); M. Petroso, versante E, prime balze venendo da Forca Resuni (Barrea), pendii rupestri, 1900 m, 28 July 2021, *F. Conti, L. Vitale s.n.* (APP No. 67016).

### 4.2. Key to the Subspecies Belonging to Pedicularis rostratospicata

1. Inflorescence with (7–)11–15(–17) flowers, calyx (6–)7–8(–10) mm long, calyx lobes (1.9–)2–2.9(–3.2) mm long—subsp. ***rostratospicata***

1. Inflorescence with (11–)18–25(–32) flowers, calyx (7.5–)9–10(–12) mm long, calyx lobes (3–)3.5–4.85(–6) mm long—**2**

2. Petiole middle leaf length (2–)8–15(–31) mm, blade middle leaf (48–)60.5–69(–80) mm long × (11–)12–17(–28) mm wide, inflorescence (53–)69.5–100(–125) mm long, corolla (13–)15–16(–16.7) mm long, beak (3.7–)4.45–5 mm long—subsp. ***helvetica***

2. Petiole middle leaf length 0(–3) mm long, blade middle leaf (33–)42–48.5(–58) mm long × (6–)8.5–12.5(–15) mm wide, inflorescence (90–)112.5–150(–175) mm long, corolla (12–)13–13.87(–15) mm long, beak (3–)3.45–3.9(–4) mm long—subsp. ***marsica***

## 5. Conclusions

In this work, we conducted a morphometric and molecular study of *Pedicularis rostratospicata*. We were able to demonstrate that the populations of this species occurring in the Central Apennines should be referred to a subspecies new to science, described here with the name *P. rostratospicata* subsp. *marsica*. Finally, we provided the conservation status assessment of the new subspecies according to IUCN categories and criteria (assessed as NT), an identification key for the three subspecies of *P. rostratospicata* (for dried herbarium specimens) and a distribution map based on the herbarium material examined.

## Figures and Tables

**Table 1:** Morphological characters studied.

Abbreviation	Description of the Character	Type
H	Height (cm)	quantitative continuous
PBLL	Petiole length of basal leaf (mm)	quantitative continuous
BBLL	Blade length of basal leaf (mm)	quantitative continuous
BBLW	Blade width of basal leaf (mm)	quantitative continuous
PMLL	Petiole length of middle cauline leaf (mm)	quantitative continuous
BMLL	Blade length of middle cauline leaf (mm)	quantitative continuous
BMLW	Blade width of middle cauline leaf (mm)	quantitative continuous
PULL	Petiole length of upper cauline leaf (mm)	quantitative continuous
BULL	Blade length of upper cauline leaf (mm)	quantitative continuous
BULW	Blade width of upper cauline leaf (mm)	quantitative continuous
IL	Inflorescence length (mm)	quantitative continuous
IBIL	Length of basal internode of inflorescence (mm)	quantitative continuous
NF	Number of flowers	quantitative discrete
BL	Bract length (mm)	quantitative continuous
CL	Calyx length (mm)	quantitative continuous
CLL	Calyx lobe length (mm)	quantitative continuous
COL	Corolla length (mm)	quantitative continuous
NL	Beak length (mm)	quantitative continuous
SS	Shape of calyx lobe (0: entire; 1: toothed)	qualitative
IH	Hairiness of inflorescence (0: sparsely hairy; 1: densely hairy)	qualitative

**Table 2:** *Pedicularis rostratospicata* populations sampled for the present AFLPseq fingerprinting study with information on localities and voucher specimens.

Sample	Taxon	Locality	Collectors	Voucher Specimens
A1285	*P. rostratospicata* subsp. *rostratospicata*	I, Trentino-Alto Adige, Colle Isarco, Val di Fleres.	*Conti & Bartolucci s.n.*	APP 66183
A1286	*P. rostratospicata* subsp. *rostratospicata*	I, Trentino-Alto Adige, Colle Isarco, Val di Fleres.	*Conti & Bartolucci s.n.*	APP 66185
A1287	*P. rostratospicata* subsp. *rostratospicata*	I, Trentino-Alto Adige, Colle Isarco, Val di Fleres.	*Conti & Bartolucci s.n.*	APP 66186
A1288	*P. rostratospicata* subsp. *helvetica*	I, Valle d’Aosta, Courmayeur, Val Veny.	*Conti & Bartolucci s.n.*	APP 65463
A1289	*P. rostratospicata* subsp. *helvetica*	I, Valle d’Aosta, Courmayeur, Val Veny.	*Conti & Bartolucci s.n.*	APP 65465
A1290	*P. rostratospicata* subsp. *helvetica*	I, Valle d’Aosta, Courmayeur, Val Veny.	*Conti & Bartolucci s.n.*	APP 65466
A1291	*P. rostratospicata* subsp. *helvetica*	I, Valle d’Aosta, Gran San Bernardo.	*Bovio, Broglio & Ganz s.n.*	APP 66182
A1292	*P. rostratospicata* subsp. *marsica*	I, Abruzzo, Serra delle Gravare (Opi), M. Irto—S. Nicola.	*Conti & Serafini s.n.*	APP 48238
A1293	*P. rostratospicata* subsp. *marsica*	I. Abruzzo, Opi, Mt. Marsicano.	*Conti & Stinca s.n.*	APP 66177
A1294	*P. rostratospicata* subsp. *marsica*	I. Abruzzo, Opi, Mt. Marsicano.	*Conti & Stinca s.n.*	APP 66179
A1295	*P. rostratospicata* subsp. *marsica*	I. Abruzzo, Opi, Mt. Marsicano.	*Conti & Stinca s.n.*	APP 66180

**Table 3:** Comparison between ROS, HEL, and MAR of characters used in the morphometric analyses. Quantitative continuous characters are reported as mean ± standard deviation and 25–75 percentiles (extreme values in brackets). For quantitative discrete characters, 25–75 percentiles are given (extreme values in brackets).

Abbreviation of Characters	ROS	HEL	MAR
H	21.64 ± 4.48 (14–)18–25(–32)	29.58 ± 4.85 (21.2–)26.47–32.65(–39.6)	30.39 ± 4.55 (25–)27–32.75(–39.3)
PBLL	33.1 ± 10.91 (17–)25.75–37.25(–54)	44.42 ± 11.31 (16–)39–52.5(–57)	28.2 ± 9.95 (15–)20.5–34(–49)
BBLL	38.5 ± 13.9 (15–)29.25–51.5(–61)	78.61 ± 15.08 (54–)67.5–85.25(–110)	55.27 ± 14.24 (30–)46.5–61(–85)
BBLW	15.12 ± 4.7 (7–)12–19(–22)	17.67 ± 5.9 (10–)14–20(–32)	16.73 ± 4.3 (10–)14–20(–25)
PMLL	10.48 ± 5.32 (7–)12–19(–22)	12.88 ± 7.97 (2–)8–15(–31)	0.47 ± 0.99 0(–3)
BMLL	36.27 ± 9.8 (16–)33–41(–59)	65.47 ± 8.78 (48–)60.5–69(–80)	45.13 ± 6.61 (33–)42–48.5(–58)
BMLW	12.57 ± 3.8 (5–)11–15(–20)	15.05 ± 4.17 (11–)12–17(–28)	10.53 ± 2.95 (6–)8.5–12.5(–15)
PULL	1.33 ± 1.88 0–2(–6)	0.1 ± 0.46 0(–2)	0.13 ± 0.52 0(–2)
BULL	12.33 ± 5.03 (14–)19–25(–38)	28.63 ± 10.3 (13–)22.5–34.5(–53)	25.6 ± 8.71 (9–)20–31(–41)
BULW	5.67 ± 2.26 (1–)4–7(–10)	6.67 ± 2.07 (3.8–)5–8(–11)	5.2 ± 2.21 (2–)3.5–6.5(–9)
IL	71.48 ± 22.4 (41–)57–80(–132)	87.05 ± 21.23 (53–)69.5–100(–125)	131.6 ± 27.04 (90–)112.5–150(–175)
IBIL	9.23 ± 3.29 (5–)7–11.4(–15)	6 ± 2.54 (3–)4–7(–10)	16.73 ± 6.53 (10–)13.5–18(–37)
NF	(7–)11–15(–17)	(11–)16–25(–32)	(16–)20–25(–29)
BL	16.52 ± 4.59 (9–)13–19(–28)	15.55 ± 3.9 (10–)13–17.5(–26)	17.67 ± 7.11 (8–)14–20(–39)
CL	7.46 ± 1 (6–)7–8(–10)	9.44 ± 1.01 (7–5–)9–10(–12)	9.47 ± 1.39 8–10.75(–12)
CLL	2.51 ± 0.43 (1.9–)2–2.9(–3.2)	3.69 ± 0.69 3–4(–5)	4.49 ± 0.74 (3.5–)4–5(–6)
COL	12.97 ± 1.23 (10.1–)12.5–13.5(–16)	15.19 ± 0.95 (13–)15–16(–16.7)	13.18 ± 0.87 (12–)13–13.87(–15)
NL	3.48 ± 0.37 3–3.8(–4)	4.65 ± 0.47 (3.7–)4.45–5	3.55 ± 0.36 (3–)3.45–3.9(–4)
SS	entire	toothed	toothed
IH	sparsely hairy	sparsely hairy	densely hairy

## Data Availability

The data presented in the current study are available within the article. Raw data of the AFLPseq fingerprinting were submitted to GenBank under the BioProject accession number PRJNA984162.
